# Effects of ambient air pollution, fresh fruit and vegetable intakes as well as maternal psychosocial stress on the outcome of newborn otoacoustic emission hearing screening

**DOI:** 10.1186/s12887-022-03328-9

**Published:** 2022-05-12

**Authors:** Bingzhi Chen, Shaoyi Chen, Lidan Duan, Muyang Zhang, Xiaoqun Liu, Yanying Duan

**Affiliations:** 1 Department of Occupational and Environmental Health, Xiangya School of Public Health, Central South University, Changsha, 410078 China; 2 Department of Children and Maternal Health, Xiangya School of Public Health, Central South University, Changsha, 410078 China

**Keywords:** Ambient air pollutant, Fresh fruit and vegetable, Life event, Hearing screening

## Abstract

**Background:**

Newborn hearing screening results indicated that more than 40% of the detected infants had no recognized risk factors. To determine whether maternal exposure to ambient air pollutants and experience of stressful life event, as well as lack of fresh fruit and vegetable during pregnancy are associated with the abnormal hearing development among newborns.

**Methods:**

A total of 1193 newborns and their mothers were recruited in this study. Personal information and covariates were collected by face to face interview. Medical examination results of newborns and their mothers were extracted from medical record. We estimated personal air pollutant exposure level through inverse distance weighted method based on data from air quality monitoring stations and assessed the auditory development of newborns via distortion product otoacoustic emission (DPOAE). Unconditional logistic regression model was used to estimate the relationship between DPOAE screening result and the potential influential factors as well as the combined effect.

**Results:**

The results indicated that PM_10_ exposure during the second trimester and stressful life event during the third trimester could increase the risk of not passing DPOAE test among newborns. However, frequent intakes of fruit and vegetable significantly reduced the risk. There was a synergetic interaction between PM_10_ exposure and stressful life event on neonatal hearing development.

**Conclusions:**

To alleviate abnormal auditory development among fetus, pregnant woman should decrease the exposures to ambient air pollutant and negative life event and at the same time, intake sufficient fresh fruit and vegetable.

## Background

Hearing impairment is one of the most common congenital anomaly at birth, constituting a serious obstacle to the developments of language and intelligence. It has also been documented to be associated with poor academic performance in school life as well as limited access to employment and social interaction in adulthood [1]. The incidence of newborn hearing loss was reported between 2 and 15% in births with abnormal conditions while 0.3% in healthy births [2, 3]. In order to alleviate the obstacle to personal development as well as the burdens on family and society, many counties in the world launch universal neonatal hearing screening project to ensure a prompt identification and rehabilitation of these infants. Meanwhile, pediatricians are dedicated to exploring and identifying the risk factors for hearing impairment in newborns, thus contributing to the effective intervention at an even earlier stage. Family history, congenital infections, craniofacial anomalies and other factors were identified successively and added to the typical risk factor list for routine screening [4]. However, a growing number of investigations found that more than 40% of the detected infants lacked these recognized risk factors or the association between these risk factors and congenital hearing impairment lacked statistical significance [4, 5].

Hearing development, containing neurosensory as well as conductive components, begins in early fetal life and becomes mature several months after birth [6]. The developmental period of hearing in utero is closely correlated to the maturity of central nervous system, which is affected by maternal environmental exposure in gestation. A recently published systematic review suggested an inverse association between maternal air pollutant exposure and head circumference at birth [7]. Also, our previous investigation indicated that the PM_2.5_ exposure in trimester 2 was negatively associated with neurological behavior score in newborns[8]. Oxidative damage and neuroinflammation were revealed by toxicological research as the main plausible mechanisms [9, 10]. In this context, prenatal exposure to air pollution may also affect auditory development of fetus, but the evidence remains limited. Besides, maternal psychological distress over pregnancy was suggested as a key adverse factor for fetal neurological development, for example, exhibiting a negative effect on fetal vision maturity [11]. It was also reported to be associated with alterations in fetal cortical gyrification, hippocampal volume and metabolite level of neurotransmitter, respectively [12]. A recent research found that antenatal maternal anxiety trait exerted an adverse effect on fetal middle cerebral artery plasticity and consequently led to poor nutrient and oxygen supply to the developing embryonic brain [13]. Therefore, maternal psychosocial condition probably play a role in the embryonic auditory development as well. Fruit and vegetable intakes during gestation were reported to be positively associated with the biparietal diameter of newborns [14]. As excellent sources of folate and vitamin C [14, 15], consumptions of fruit and vegetable are probably essential for cell division via transferring and processing one-carbon in DNA synthesis process [16], and exert a significant protective effect on vision and olfaction against ionizing radiation during neurulation in rodent model[17]. Given that vitamin C is a pivotal contributor to the redox homeostasis, deficiency of fruit and vegetable in maternal diet was probably linked to the increase of oxidative damage in the developing nervous system [18].

As there are similar biological mechanisms through which ambient air pollutant exposure, maternal stress and fruit and vegetable intakes influence the developing nervous system, combined exposure may lead to synergistic or antagonize effects. Therefore, in the present study, we enrolled a total of 1190 healthy pregnant women, and collected information via structural questionnaire and medical record, on the one hand, to elucidate the individual association of air pollutant, maternal distress and fruit and vegetable intakes with newborn hearing screening outcomes, on the other hand, to assess the potential combined effects of air pollutant, maternal distress and fruit and vegetable intakes on hearing development.

## Methods

### Study design and population

This population-based prospective investigation was conducted in Changsha city, Hunan province, which was previously reported elsewhere [8]. A total of 1190 healthy pregnant women were enrolled in this study in 2017, who had been lived in Changsha for at least 1 year and registered in Hunan Maternal and Child Health Hospital for delivery. As we focused on the effect of living environment on hearing screening result, pregnant woman with occupational exposure such as dust and excessive noise were excluded. During the routine antenatal examination, maternal life stressor, anxiety and depression were assessed by Life Events Scale of Pregnant Woman (LESPW) [19], Spielberger State-Trait Anxiety Inventory (STAI) [20] and Edinburgh Postnatal Depression Scale (EPDS), respectively [21]. LESPW contains 53 items and divides into subjective event (SE) and objective event (OE). OE is further divides into three grades, OE1, OE2 and OE3. The thresholds of SE, OE1, OE2 and OE3 were 130, 310, 225 and 1450, respectively. STAI is designed to assess state anxiety and traint anxiety through 40 items and the threshold scores were set at 39 and 41 in this investigation, respectively. EPDS contains 10 items and score equal to or higher than 9 is considered as depression. If intake frequency of fruit or vegetable was less than once a month, we defined it as no fruit or vegetable intake**.** After childbirth, we conducted a face to face questionnaire survey among the participants. The questionnaire contained basic information and potential confounding factors, including maternal age, height, weight before pregnancy, reproductive history, medical history during pregnancy, period of residence in Changsha, food intake frequent, parent's smoking habit (including passive smoking), alcohol intake and home address. Particularly, we pay attention to the factors related to indoor air pollution (kitchen fuel, house decoration during pregnancy and passive smoking) and dietary habit (intake of fruit, vegetable, milk, soy milk and tea). After finish the interview, all the participants signed the written informed consent. The research protocol and process have been approved by the ethics committee of Xiangya School of Public Health, Central South University.

### Estimation of exposure to ambient air pollutants

In this study, we used data from Changsha City Air Quality Monitoring Station to assess the maternal exposure level of air pollutants during pregnancy. There are ten monitoring stations in the urban area of ​​Changsha, recording the concentrations of five kinds of atmospheric pollutants (PM_10_, PM_2.5_, CO, NO_2_, SO_2_). Daily average concentrations of the five pollutants and Inverse Distance Weighted (IDW) were used in the estimation of individual exposure level [22]. IDW selects the concentrations from the four nearest monitoring points to the maternal home address. At the same time, satellite map is used to measure the straight-line distance (d1 to d4) from the four monitoring points to the maternal address. The specific calculation formula is as follows:

$$C=\frac{\frac{1}{{\mathrm{d}}_{1}^{2}}\times {\mathrm{c}}_{1}+\frac{1}{{\mathrm{d}}_{2}^{2}}\times {\mathrm{c}}_{2}+\frac{1}{{\mathrm{d}}_{3}^{2}}\times {\mathrm{c}}_{3}+\frac{1}{{\mathrm{d}}_{4}^{2}}\times {\mathrm{c}}_{4}}{\frac{1}{{\mathrm{d}}_{1}^{2}}+\frac{1}{{\mathrm{d}}_{2}^{2}}+\frac{1}{{\mathrm{d}}_{3}^{2}}+\frac{1}{{\mathrm{d}}_{4}^{2}}}$$

### Hearing screening for newborns

Distortion product otoacoustic emission (DPOAE) was employed to assess the auditory development of newborns via otoacoustic emission screening instrument (GSI70, USA). Parameters were set as following: f2 / f1 = 1.22, stimulus intensity L1 = 65 dB SPL, L2 = 55 dB SPL, frequency range (represented by f2) f2 = 1.5, 2.0, 3.0, 4.0, 5.0, 6.0 kHz, average time = 4 s. Inspection process was carried out in a soundproof shielded room when newborn was in a quiet state. Before screening, investigator should confirm that the newborn's external auditory meatus is free of foreign matter, and then select the suitable probe adapting to the newborn's external auditory. The instrument displays the result as PASS or NOT PASS. Both ears of the newborn are subject to DPOAE test, and as long as one ear fails the test, it is recognized as not passed. This screening is carried out by a well-trained investigator who does not know about the information of newborn. A warm and quiet area was chosen for screening test and before test, investigator examine the external auditory canalfor debris, wax etc.

### Statistical analysis

Continuous variables were expressed as mean ± standard deviation, and categorical variables were expressed in terms of frequency and percentage. A total of 1190 newborns were divided into group "PASS" and group "NOT PASS" according to the DPOAE screening result. Chi-square test and Mann–Whitney U test were used to compare the distribution differences of diverse variables between two groups.

Logistic regression model was used for estimation the relationship between DPOAE screening result and the potential influential effecters. In the univariate analysis, only the exposure levels of PM_10_ and PM_2.5_ were found to be statistically associated with screening outcome among five air pollutants, Therefore, we only included the levels of PM_10_ and PM_2.5_ in the form of quartile into multivariable model, which was adjusted by maternal age, paternal age, maternal educational level, premature delivery, newborn sex, birthweight, instrument assist in child delivery and amnionic fluid status. And the results were displayed as odds ratio (OR) and 95% confidence interval (95% CI).

In the interaction analysis, the concentrations of PM_10_ and PM_2.5_ were converted into low and high groups by the corresponding medians, and low exposure group was used as reference. Logistic regression model was applied to estimate the association of screening result with fruit or vegetable intake, mental stimulation and parity respectively after stratification. We established an interaction term in the analysis of overall data and gave the *p* value as interaction *p* value.

All analyses were performed using SPSS 22.0. The estimated value, its 95% CI and *p* value were given in the results, and P < 0.05 was considered statistically significant.

## Results

Among the 1190 newborns, 166 (13.9%) did not pass the DPOAE test. The distribution and statistical analysis results of the variables in the not pass and pass group were shown in Table 1. We observed that the differences of parity, stressful life event during pregnancy, and prenatal exposure to PM_10_ and PM_2.5_ were statistically different between the two groups (*P* < 0.05), which may be potential risk factors for newborn not passing DPOAE test. As intake frequencies of fruit and vegetable less than once a month and experience of stressful life event were rare in real life situation, the number of pregnant woman with these characteristics was relatively small.

**Table 1:** Demographic information, covariates and personal air pollutant exposure level between pass and not pass of DPOAE test groups (*n* = 1190)

	**Not pass(n = 166)**	**Pass(n = 1024)**	***p***
**Maternal age (years)**			0.245
23	3(1.8)	26(2.5)	
23–30	103(62.0)	564(55.2)	
≥ 30	60(36.1)	432(42.3)	
**Maternal education**			0.883
Middle school or below	5(3.0)	28(2.7)	
High school	28(16.9)	159(15.5)	
College or above	133(80.1)	837(81.7)	
**Parity**			**0.049**
Primiparous	114(69.9)	628(61.9)	
Multiparous	49(30.1)	386(38.1)	
**Paternal age(years)**			0.199
< 30	67(40.4)	341(33.4)	
30–35	61(36.7)	433(42.4)	
≥ 35	38(22.9)	248(24.3)	
**Neonates gender**			0.210
Male	96(57.8)	537(52.6)	
Female	70(42.2)	484(47.4)	
**Midwifery**			0.730
Yes	33(19.9)	192(18.8)	
No	133(80.1)	832(81.3)	
**Gestational age(weeks)**			1.000
37	162(97.6)	999(97.7)	
37–42	4(2.4)	24(2.3)	
**Birth weight(g)**			0.460
2500	0(0.0)	8(0.8)	
2500–3950	159(95.8)	980(95.8)	
≥ 4000	7(4.2)	35(3.4)	
**Amniotic fluid condition**			0.556
Normal	133(80.1)	816(79.8)	
Amniotic fluid turbidity	30(18.1)	193(18.9)	
Oligohydramnios	0(0.0)	5(0.5)	
Umbilical cord around the neck	3(1.8)	9(0.9)	
**Folate intake**			0.306
No	8(4.8)	71(7.0)	
Yes	158(95.2)	950(97.0)	
**Fruit or vegetable intake**			0.126
No	7(4.2)	20(2.0)	
Yes	159(95.8)	1001(98.0)	
**Milk or soy milk intake**			0.873
No	47(28.3)	295(28.9)	
Yes	119(71.7)	725(71.1)	
**Family relationship**			0.987
Well	139(85.8)	862(85.0)	
General	19(11.7)	123(12.1)	
Worse	3(1.9)	23(2.3)	
Terrible	1(0.6)	6(0.6)	
**Stress and anxiety**			0.518
No	118(71.1)	751(73.5)	
Yes	48(28.9)	271(26.5)	
**Stressful life event**			**0.021**
No	155(93.4)	987(97.0)	
Yes	11(6.6)	31(3.0)	
**House renovation**			0.660
No	163(98.2)	992(97.3)	
Yes	3(1.8)	28(2.7)	
**Kitchen fuel**			0.355
Fuel gas	143(86.1)	907(88.7)	
Coal	16(9.6)	90(8.8)	
Electric	4(2.4)	19(1.9)	
Others	3(1.8)	6(0.6)	
**Maternal active smoking(cigarettes per day)**			0.796
Never	165(99.4)	1013(98.9)	
Less than 10	1(0.6)	9(0.9)	
10–20	0(0.0)	2(0.2)	
**Paternal active smoking(cigarettes per day)**			0.733
Never	102(61.4)	640(62.5)	
Less than 10	43(25.9)	231(22.6)	
10–20	18(10.8)	128(12.5)	
More than 20	3(1.8)	25(2.4)	
**Passive smoking**			0.821
No	119(72.6)	748(73.4)	
Yes	45(27.4)	271(26.6)	
**Maternal alcohol intake**			0.134
Never	157(94.6)	989(96.9)	
Rarely	9(5.4)	32(3.1)	
**Paternal alcohol intake**			0.758
Never	99(59.6)	607(59.3)	
Rarely	61(36.7)	389(38.0)	
Often	6(3.6)	27(2.6)	
**PM**_**10**_**(μg/m3)**	70.20 ± 7.24	68.27 ± 6.69	**0.001**
**PM**_**2.5**_**(μg/m3)**	44.37 ± 6.18	43.05 ± 5.49	**0.017**
**CO(mg/m3)**	1.00 ± 0.09	0.99 ± 0.10	0.070
**NO**_**2**_**(μg/m3)**	36.37 ± 4.38	35.83 ± 4.24	0.196
**SO**_**2**_**(μg/m3)**	13.97 ± 1.67	13.86 ± 1.53	0.467

Table 2 summarizes the average exposure concentrations of PM_10_, PM_2.5_, CO, NO_2_ and SO_2_ among pregnant women in the first, second and third trimesters. The first trimester had the highest exposures level of the five air pollutants and the corresponding average levels were 80.58 μg/m, 49.15 μg/m3, 1.05 mg/m3, 40.17 μg/m3 and 14.56 μg/m3. While the second trimester had the lowest exposure levels of air pollutants and the average levels were PM_10_ 57.31 μg/m, PM_2.5_ 33.34 μg/m3, CO 0.94 mg/m3, NO_2_ 30.82 μg/m3 and SO_2_ 13.59 μg/m3.

**Table 2:** Descriptive statistics for air pollution during different time windows attributed to pregnancy women (*n* = 1190)

Variables	Mean ± SD	Min	P_25_	P_50_	P_75_	Max	IQR
**The first trimester**							
PM_10_(μg/m^3^)	80.58 ± 10.86	49.78	72.76	81.94	88.43	106.91	15.67
PM_2.5_(μg/m^3^)	49.15 ± 11.31	24.31	40.02	47.82	56.01	81.91	15.99
CO(mg/m^3^)	1.05 ± 0.16	0.66	0.92	1.04	1.16	1.49	0.23
NO_2_(μg/m^3^)	40.17 ± 8.40	9.53	33.79	41.22	46.34	59.83	12.55
SO_2_(μg/m^3^)	14.56 ± 2.62	6.38	12.4	14.36	16.57	21.16	4.17
**The second trimester**							
PM_10_(μg/m^3^)	57.31 ± 13.72	36.37	47.58	52.3	63.11	104.3	15.53
PM_2.5_(μg/m^3^)	33.34 ± 12.65	19.29	25.77	28.03	32.1	72.13	6.33
CO(mg/m^3^)	0.94 ± 0.13	0.7	0.85	0.93	1.02	1.57	0.17
NO_2_(μg/m^3^)	30.82 ± 6.84	15.68	26.4	29.95	33.61	57.07	7.21
SO_2_(μg/m^3^)	13.59 ± 1.59	9.36	12.67	13.73	14.6	20.06	1.93
**The third trimester**							
PM_10_(μg/m^3^)	68.63 ± 15.37	40.4	55.33	68.18	80.06	116.65	24.73
PM_2.5_(μg/m^3^)	48.23 ± 15.02	19.77	34.07	48.32	60.77	85.72	26.7
CO(mg/m^3^)	1.00 ± 0.28	0.67	0.82	0.92	1.08	2.86	0.26
NO_2_(μg/m^3^)	37.15 ± 8.32	17.85	30.98	37.69	42.81	60.62	11.83
SO_2_(μg/m^3^)	13.51 ± 1.63	9.79	12.32	13.53	14.38	20.31	2.07

Association between exposures to atmospheric pollutants during pregnancy and DPOAE test results of newborns was estimated by univariable and multivariable logistic regression analysis and the results were displayed in Table 3. The concentration of PM_10_ in the second trimester significantly increased the risk of losing the test with crude OR of 1.305 (95% CI, 1.117, 1.524), which was robust to the adjustment of parental age, maternal education level, premature, sex, birthweight, dietary habit, anxiety, depression, life event, indoor air pollution and et al. As for PM_2.5_, the level in the second trimester was significantly associated with increased risk of not passing DPOAE test with crude OR of 1.207 (95% CI, 1.028, 1.417). However, the significance alleviated into null after adjustment of potential covariables.

**Table 3:** Odds ratio (95% CI) of not passing DPOAE test for exposure to PM_10_ and PM_2.5_ during different time windows (*n* = 1190)

	**Unadjusted**	**Model 1**	**Model 2**	**Model 3**
**PM**_**10**_				
1st trimester	1.060(0.886,1.267)	1.177(0.932,1.487)	1.212(0.956,1.538)	1.203(0.941,1.538)
2nd trimester	1.305(1.117,1.524)**	1.537(1.170,2.019)**	1.517(1.149,2.004)**	1.541(1.162,2.043)**
3rd trimester	1.113(0.935,1.325)	0.850(0.599,1.206)	0.845(0.594,1.203)	0.846(0.591,1.211)
**PM**_**2.5**_				
1st trimester	1.047(0.768,1.425)	0.929(0.645,1.338)	0.853(0.586,1.241)	0.837(0.571,1.227)
2nd trimester	1.207(1.028,1.417)*	0.919(0.712,1.186)	0.947(0.730,1.227)	0.963(0.741,1.253)
3rd trimester	1.134(0.837,1.535)	1.369(0.913,2.053)	1.359(0.897,2.060)	1.324(0.867,2.020)

Levels of PM_10_ and PM_2.5_ were in the form of quartile in this multivariable model. Model 1 adjusted parental age, maternal education level, premature, sex, birth weight during each time window. Model 2 adjusted dietary habit, anxiety, depression, life event during each time window based on Model 1. Model 3 adjusted indoor air pollution during each time window based on Model 2

^*^p ≤ 0.05

^**^p ≤ 0.001

As Table 4 showed, indoor air pollution including smoking, house renovation and kitchen fuel seemed to have no evident effect on the risk of losing the screening test.

**Table 4:** Odds ratio (95% CI) of not passing DPOAE test for exposure to indoor air pollution during the whole pregnancy period (n = 1190)

	**Unadjusted**	**Model 1**	**Model 2**	**Model 3**
**Maternal active smoking**	0.558(0.072,4.352)	0.482(0.061,3.823)	0.521(0.065,4.154)	0.463(0.055,3.906)
**Passive smoking**	1.044(0.721,1.511)	1.045(0.710,1.536)	0.932(0.623,1.393)	0.788(0.516,1.201)
**House renovation**	0.652(0.196,2.170)	0.715(0.210,2.439)	0.697(0.202,2.403)	0.696(0.198,2.449)
**Kitchen fuel**				
Fuel gas	REF	REF	REF	REF
Coal	1.128(0.644,1.975)	1.162(0.652,2.071)	0.920(0.441,1.920)	0.777(0.346,1.742)
Electric	1.335(0.448,3.982)	1.262(0.414,3.845)	1.244(0.406,3.809)	1.356(0.437,4.211)
Others	3.171(0.784,12.823)	3.763(0.817,17.332)	2.801(0.596,13.153)	2.917(0.589,14.439)

Model 1 adjusted parental age, maternal education level, premature, sex, birth weight during each time window

Model 2 adjusted dietary habit, anxiety, depression, life event during each time window based on Model 1

Model 3 adjusted PM_10_, PM_2.5_ during each time window based on Model 2

^*^p ≤ 0.05

^**^p ≤ 0.001

Newborns with mother experiencing stressful life event during the 3rd trimester had more than 3 times higher risk of not passing DPOAE test with crude OR of 3.568 (95% CI, 1.266, 10.055) and adjusted OR of 3.217 (95% CI, 1.080, 9.581), 3.366 (95% CI, 1.097, 10.330), 3.678 (95% CI, 1.183, 11.433) in different adjusted models (Table 5). Conversely, pregnant mother with regular intake of fruit and vegetable would decrease the risk of not passing the test and the association remained significant after adjustment of parental age, maternal education level, premature, parity, sex, birth weight, amniotic fluid condition, PM _2.5_ and PM_10_.

**Table 5:** Odds ratio (95% CI) of not passing DPOAE test for dietary habits and stressful life event during pregnancy period (n = 1190)

	**Unadjusted**	**Model 1**	**Model 2**	**Model 3**
**Folate intake**	1.476(0.697,3.125)	1.563(0.718,3.405)	1.418(0.643,3.129)	1.420(0.640,3.151)
**Fruit or vegetable intake**	0.454(0.189,1.091)	0.338(0.133,0.860)*	0.350(0.135,0.904)*	0.387(0.147,1.017)
**Milk or soy milk intake**	1.030(0.716,1.482)	1.006(0.683,1.481)	0.961(0.644,1.433)	0.951(0.635,1.423)
**Tea intake**	1.167(0.779,1.750)	1.114(0.722,1.721)	1.144(0.709,1.845)	1.297(0.745,2.260)
**Stress and anxiety**	1.127(0.784,1.621)	1.013(0.684,1.500)	0.955(0.637,1.433)	0.977(0.662,1.501)
**Stressful life event**				
1st trimester	0.889(0.196,4.043)	0.920(0.197,4.301)	1.079(0.228,5.115)	1.068(0.223,5.118)
2nd trimester	2.861(0.830,9.870)	2.764(0.757,10.087)	2.944(0.772,11.226)	3.253(0.839,12.609)
3rd trimester	3.568(1.266,10.055)*	3.217(1.080,9.581)*	3.366(1.097,10.330)*	3.678(1.183,11.433)*

Model 1 adjusted parental age, maternal education level, premature, sex, birth weight during each time window

Model 2 adjusted PM_10_, PM_2.5_ during each time window based on Model 1

Model 3 adjusted indoor air pollution during each time window based on Model 2

^*^p ≤ 0.05

^**^p ≤ 0.001

After stratification by the medians of PM_2.5_ or PM_10_, we found that frequent intake of fruit and vegetable significantly reduced the risk of losing DPOAE test only among mother with low exposure level of PM_2.5_ and PM_10_. Stressful life event during pregnancy significantly increased the risk of not passing DPOAE test by 4 time sonly for newborns with higher prenatal exposure level of PM_10_ (OR, 4.242, 95% CI, 1.742, 10.329, interaction *p* value = 0.033) (Table 6).

**Table 6:** Combined effects of air pollutant and other risk factors on the results of DOPAE test among newborns (*n* = 1190)

	**PM**_**10**_			**PM**_**2.5**_		
	**Low**	**High**	**Interaction *p***	**Low**	**High**	**Interaction *p***
**Parity**						
Primiparous	REF	REF	-	REF	REF	-
Multiparous	0.489(0.233,1.028)	0.725(0.409,1.285)	0.602	0.616(0.313,1.210)	0.547(0.296,1.010)	0.918
**Fruit or vegetable intake**						
No	REF	REF	-	REF	REF	-
Yes	0.212(0.059,0.763)*	0.592(0.149,2.346)	0.405	0.243(0.066,0.887)*	0.485(0.118,1.993)	0.383
**Stressful life event**						
No	REF	REF	-	REF	REF	-
Yes	0.364(0.044,2.994)	4.242(1.742,10.329)**	**0.033**	3.549(1.125,11.202)*	1.749(0.656,4.663)	0.515

Model adjusted all the covariates shows in Table 3, Table 4 and Table 5

Interaction p-value was indicated as the significance of the associations air pollution exposure level and other risk factors on not passing DOPAE

Interaction p-value < 0.1 was indicated as statistical significance

^*^p ≤ 0.05

^**^p ≤ 0.001

## Discussion

Our results show that PM_10_ exposure during the second trimester and stressful life event during the third trimester could increase the risk of not passing DPOAE test among newborns, while frequent intakes of fruit and vegetable significantly reduced the risk. Notably, a synergetic interaction was found between PM_10_ exposure and stressful life event on neonatal hearing development. To the best of our knowledge, this is the first epidemiological study to elucidate the effects of PM_10_, stressful life event and intakes of fruit and vegetable during pregnancy on auditory development among human fetus.

PM_10_ is known as a kind of inhalable particle with aerodynamic diameter of 10 microns or less, and it can enter the human respiratory tract, inducing systematic inflammation [23] and oxidative damage to both mother and fetus[24]. Evidence related to the adverse effect of prenatal exposure of PM_10_ on auditory development is limited. However, a couple of epidemiological investigation consistently reported the positive association between prenatal exposure to tobacco smoke and risk of hearing impairment among children or adolescent [25, 26]. It is worthy noting that PM_10_ has a large surface area and can absorb a variety of harmful substances, such as polychlorinated biphenyls (PCBs), dioxins and heavy metals [27, 28]. PCBs are considered as neurotoxic substances and could incur abnormal hearing development [29–33]. A study from central Taiwan reported that newborns had an increased risk of raising low-frequency hearing threshold due to exposure to PCBs from contaminated rice oil [29].Dioxin is a ubiquitous persistent environmental pollutant and in animal model, it increased cochlear sensitivity threshold at 1.5 months of age due to low dose exposure in embryonic stage [34]. Therefore, particulate matter itself and the adsorbed component could both contribute to the impairment of hearing development among newborns.

Negative life event during pregnancy could elicit stress status among pregnant woman, which is a systemic non-specific adaptive response and associated with increased incidence of brain development disorder in offspring [35] as well as pregnancy complication among pregnant woman [36, 37]. Investigators found that psychological trauma experienced in pregnancy, for example, destruction of the World Trade Center in 2001, was negatively associated with newborn head circumference, implying the potential influence on fetal neurological development [38, 39]. In rodent model, prenatal stress was reported eliciting diversity of neuropsychological impairments among offspring, which was due to the disturbance in early brain development programming [40]. Notably, Kadner and colleagues found that exposure of pregnant rats to prenatal stress could increase the low-frequency hearing threshold of male offspring, probably through accelerating cochlear degeneration and/or disrupting cochlear development [41]. In line with the above-mentioned research, we observed a positive association of prenatal stressful life event with risk of failure in auditory screening test. However, few study elucidated the underlying mechanism. A growing number of literatures suggested the possible involvement of oxidative stress in the association between maternal psychological stress and fetal hearing impairment [42, 43]. Oxidative damage was one of the most widely accepted pathogenic mechanism underlying diverse birth deficits or disorder, and played a key role in hearing impairment caused by heavy metal and noises [44, 45]. In this context, oxidative stress could be one of the plausible mechanisms for the adverse effect of prenatal psychological stress on fetal auditory development.

Hearing loss usually occur accompanied with folic acid-deficient condition [46]. At the same time, folic acid supplementation can improve the outcome of hearing loss, which is related to the preventions of inner ear collagen deposition and oxidative stress [47]. In addition, vitamin C, a kind of non-enzymatic antioxidants, exerted a protective effect against impairment in hearing threshold induced by ototoxic drugs in experimental mice [48] and in epidemiological study, it was negatively correlated to hearing impairment among both diabetes patient and control group [49]. In the present study, consumption of fruit and vegetable during pregnancy could increase the pass rate of DPOAE, which may be related to the protective effects of folic acid and vitamin C in fruit and vegetable. In analysis of interaction, we found that in PM_10_ and PM_2.5_ low exposure groups, intake of fruit and vegetable during pregnancy showed a significant protective effect for hearing development while this effect alleviated to null in high exposure group. It may be due to the higher level of oxidative stress in the PM_2.5_ and PM_10_ high-exposed group, which could not be reversed by the antioxidant component in fruit and vegetable. Also, experience of stressful life event during pregnancy significantly increased higher risk of losing DPOAE among newborns with high level of prenatal PM_10_ exposure as compared to the low exposed group, which was probably attributed to the synergetic oxidative damages from both stressful life event and high level of PM_10_ exposure.

A growing number of literatures reported the adverse effect of smoking and passive smoking on hearing impairment among fetus, children and adults [50–52], which was in line with the result from animal research [53]. However, in our study, we failed to find a correlation between cigarette exposure and DPOAE result, which may be due to the extremely low proportion of mother who were exposed to second-hand smoke or smoked during pregnancy.

In the present study, we applied otoacoustic emission (OAE) screening test to detect infants with hearing impairment. OAE originates from normal cochlear activity and can also be inspired by sound stimulation, which releases outwards through ossicular chain, tympanic membrane and external auditory meatus. The signal can be detected by instrument and any abnormalities in ossicular chain, tympanic membrane and external auditory meatus will result in fail in screening test. OAE examination is the most commonly used method of newborn hearing screening in pediatric clinics, and it has the advantages of rapid, accurate and non-invasive [54, 55]. A total of 13.4% participants in the present study did not pass the test, which was much higher than the results from Singapore [56] and UK [57], but lower than the screening outcome from India [58]. As a neonatal hearing screening tool, optoacoustic emissions along with auditory brainstem responses (ABRs) could not perform without error. Although, more reliable frequency range was applied in the present study, the false positive rate was estimated to be 8%according to published literature [59, 60]. Therefore, diagnostic hearing impairment is warranted in the following study to confirm these findings.

Besides, this study has other limitations. Personal exposure to ambient air pollutant was estimated via air monitoring data from government websites by IDW method, without considering the influence of activity pattern, which would misclassified the exposure grades. Besides, the concentrations of PCBs, dioxins and heavy metals in PM_10_ were not be measured in this study.

## Conclusions

The present investigation indicated that PM_10_ and stressful life event during pregnancy would increase the risk of not passing newborn otoacoustic emission hearing screening. However, sufficient consumptions of fruit and vegetable could reduce the risk. In addition, there was a synergetical effect between PM_10_ and stressful life event on neonatal hearing development. Therefore, to alleviate abnormal auditory development of fetus, pregnant woman should avoid exposure to ambient air pollutant and experience of negative life event and at the same time, intake sufficient fresh fruit and vegetable.

## Data Availability

The datasets used and analysed in the current study were available from the corresponding author on reasonable request.

## Abbreviations

LESPW: Life Events Scale of Pregnant Woman
STAI: Spielberger State-Trait Anxiety Inventory
EPDS: Edinburgh Postnatal Depression Scale
DPOAE: Distortion product otoacoustic emission
